# Complex Primary Total Knee Arthroplasty in a Young Patient With Neurofibromatosis Type One and Multidirectional Knee Instability: Technical Tips and Outcome

**DOI:** 10.7759/cureus.39721

**Published:** 2023-05-30

**Authors:** Eustathios Kenanidis, Ekaterini Klonou, Ioannis Leonida, Eleftherios Tsiridis

**Affiliations:** 1 Papageorgiou General Hospital, Aristotle University of Thessaloniki, School of Medicine, Thessaloniki, GRC; 2 Centre for Orthopaedic and Regenerative Medicine (CORE) Centre for Interdisciplinary Research and Innovation (CIRI), Aristotle University of Thessaloniki, School of Medicine, Thessaloniki, GRC

**Keywords:** neurofibromatosis type 1 (nf-1), neurologic disorder, primary total knee arthroplasty, complex primary knee arthroplasty, multidirectional knee instability, rotating hinged, young patient, tka, total knee arthroplasty, neurofibromatosis

## Abstract

Neurofibromatosis is an inherited disorder that causes skin discoloration and tumors. The musculoskeletal symptoms are specific, including bone deformities, dysplasia, joint instability, and osteoporosis. We present a rare case of a young patient with neurofibromatosis and multidirectional knee instability who underwent a successful complex primary knee replacement surgery. Stress right knee radiographs showed global joint instability with permanent anterior knee dislocation, excessively hypoplastic femoral condyles and patella, joint surfaces incongruency, and hypoplastic varus tibia, with intraluminal midshaft bone bridge causing severe stenosis. The patient could not walk, had an unstable recurvatum right knee, and used a wheelchair for her professional activities. The surgery involved a fully cemented rotating-hinged total knee arthroplasty with tibial and femoral stems. After three years of follow-up, the patient remains pain-free, fully ambulatory with no walking aids, a stable knee, a full range of motion, and no signs of aseptic loosening. This case highlights the decision-making difficulties and the significant surgical challenges faced during the operation.

## Introduction

Neurofibromatosis is a genetic disorder that causes the growth of non-cancerous tumors on the skin’s nerve sheaths, known as neurofibromas [1]. There are three types of neurofibromatosis, with type one being the most prevalent [2]. Neurofibromatosis type one, also called von Recklinghausen’s disease, is a hereditary disorder that affects approximately 1 per 3,000 newborns worldwide [3]. The gene responsible for neurofibromatosis type one produces neurofibromin, which regulates cell growth [1,2].

The diagnosis of neurofibromatosis type one is typically based on clinical observations. Common symptoms include various pigmented skin spots, the so-called café-au-lait macules, freckling, neurofibromas, and optic gliomas [4]. Patients with type one neurofibromatosis are often intelligent but may have learning and behavioral difficulties [4]. Musculoskeletal manifestations are limited but may include reduced muscle size and function, bone deformities, and a higher risk of fractures [4,5]. Giant neurofibromas can cause deformities in ribs, pelvis, and long bones [5]. Early-onset scoliosis and spinal abnormalities are also possible. Bone growth abnormalities and tibial fractures can lead to malunion and poor healing [4,5].

## Case presentation

A 30-year-old white Caucasian female presented to the orthopedic clinic with a long history of right knee instability and severe edema. The patient had limited pain during weight-bearing but progressively deteriorating knee instability, making weight-bearing impossible without support. She could only stand or walk using a walking aid and knee brace for a short walk. She had been diagnosed with type one neurofibromatosis at the age of four during a diagnostic evaluation of leg length discrepancy caused by the longer right limb. She had a family history of neurofibromatosis and no history of diabetes mellitus.

The patient had undergone three surgical operations in the past. During the first two operations, which took place at the age of four years, the surgeons aimed to remove the knee neurofibroma and guide growth to manage the leg length discrepancy, performing temporary hemiepiphysiodesis on the right knee. Knee implants were removed at 12 years. Since then, the neurofibroma in the front of the knee had been exerting pressure and causing deformation of the bone and ligament structures underneath. This led to the underdevelopment of the distal femur, patella, and proximal tibia, resulting in incongruent joint surfaces and progressing multidirectional instability. As a result, Charcot neuropathy had developed. The neurovascular status of the patient was intact. A third procedure to partially remove the neurofibroma tumor in the lateral aspect of the knee and an attempt to stabilize the knee using a hamstring autograft to reconstruct the medial collateral knee ligament was performed at the age of 18 without success.

**Figure 1:**
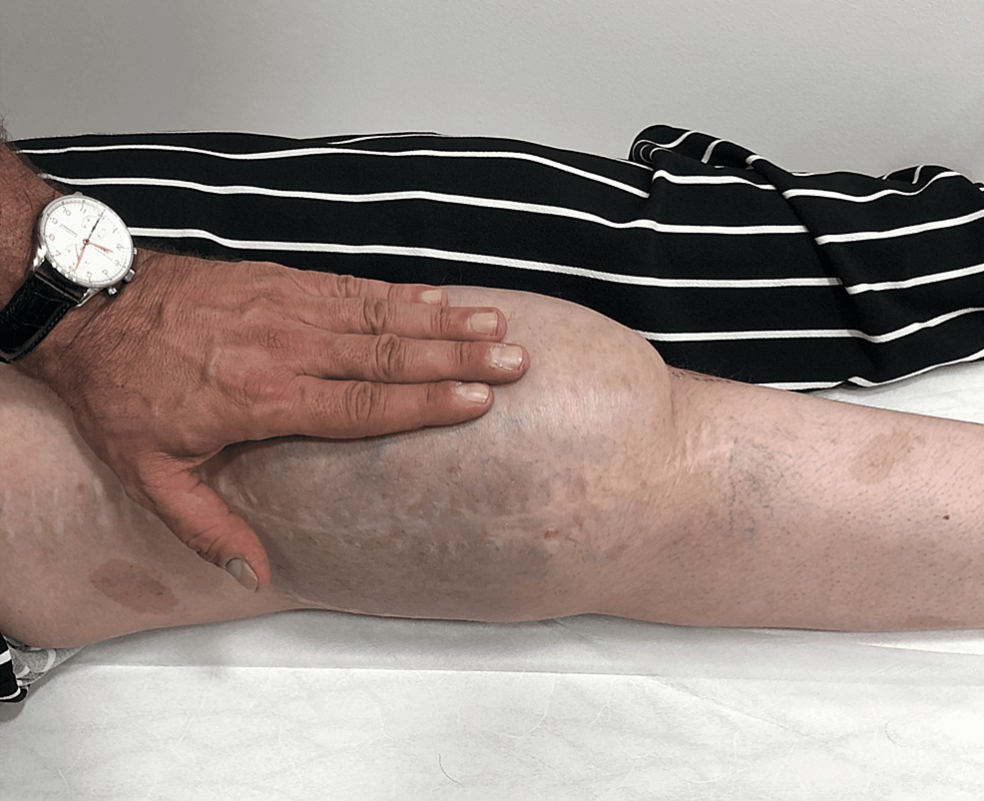
Image shows the side view of the patient’s knee before surgery. There is noticeable swelling on the front and outer side of the knee. It is important to observe the doctor touching and examining a neurofibroma on the knee.

The patient had multiple café-au-lait spots on her body and limbs during the physical examination. Additionally, there was significant swelling on the anterolateral side of her knee (Figure 1). There were no other issues in the neighboring joints.

The right knee had extreme multidirectional instability and no limitation in passive and active range of motion (ROM); however, with profound tibiofemoral and patella-femoral incongruency and recurvatum deformity (Video 1).

**Video 1:** A preoperative clinical examination of the patient’s left knee demonstrated significant swelling on the anterolateral side of the knee, extreme multidirectional instability, no limitation in passive and active range of motion, and profound tibiofemoral and patella-femoral incongruency. In addition, the doctor recognized the size of the patella and joint line.

The patient was unable to actively raise the leg. There were no other issues with the contralateral left knee. The patient had muscle weakness due to prolonged immobilization but did not experience any knee pain. Due to gross deformity and apparent knee instability, the patient could not walk alone or perform daily activities unless using a wheelchair. The Oxford Knee Score was 16.

**Figure 2:**
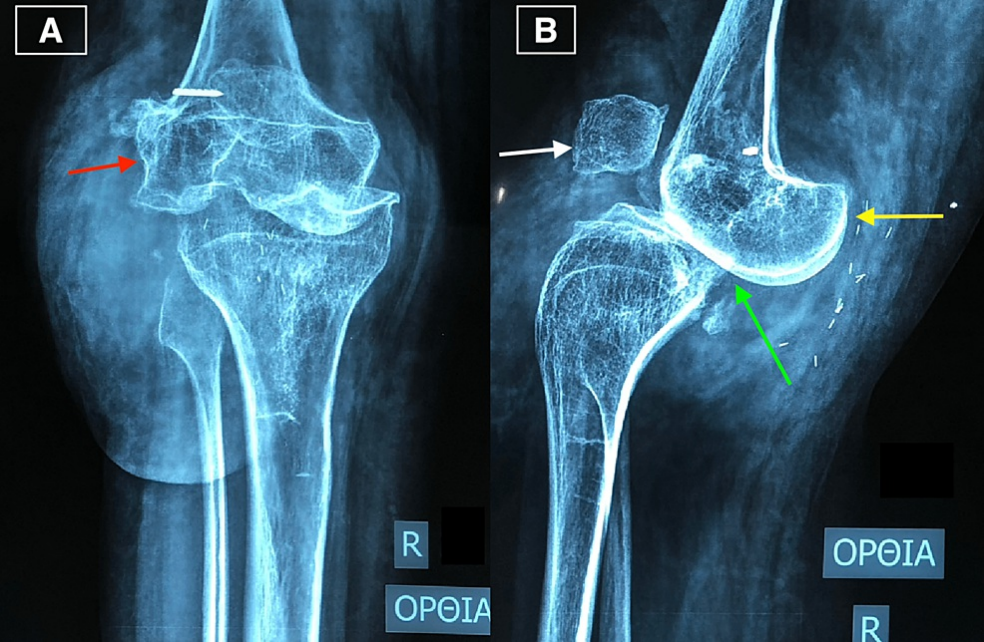
Preoperative (A) anteroposterior and (B) lateral stress knee radiographs showing the hypoplastic lateral femoral condyle (red arrow), patella (white arrow), and distal femur; the hypertrophy of posterior femoral condyles (yellow arrow); the anterior knee dislocation; and joint surfaces incongruency (green arrow). Additionally, there is diffuse osteoporosis with thin femoral and tibial cortices, early knee osteoarthritis, and clips from previous knee surgeries.

After conducting magnetic resonance imaging, it was discovered that the patient had a large neurofibroma on the anterolateral aspect of the right knee. Stress radiographs also showed that the patient had a hypoplastic lateral femoral condyle, patella, and distal femur and hypertrophy of posterior femoral condyles, increasing the anteroposterior dimension of the distal femur, thus influencing the flexion gap of the knee, as well as posterior femoral dislocation to the tibia and joint surfaces incongruency. In addition, the patient had diffuse osteoporosis with thin femoral and tibial cortices and early knee osteoarthritis (Figure 2).

During the consultation, the following treatment options were recommended to the patient: protected weight-bearing with a brace and crutches, knee arthrodesis, or constrained knee arthroplasty. The patient declined the conservative treatment and became aware of the advantages and disadvantages of arthrodesis. As a result, a rotating-hinged total knee arthroplasty (RH-TKA) was chosen as the best surgical option.

The patient underwent a right RH-TKA (NexGen® Rotating Hinge Knee, Zimmer Biomet), which limits the knee’s side-to-side (mediolateral) and front-to-back (anteroposterior) movement while allowing for flexion, extension, and rotation between the femoral and tibial components. We aimed for mechanical alignment of the knee as the only option for RH-TKA. The surgery was performed using the previous skin approach through a medial parapatellar approach. The anterior knee neurofibrous tissue was found densely adherent to the underlying capsule during the procedure. Most of this tissue was removed to avoid harming the skin and capsule. The distal femur and proximal tibia were adequately exposed, revealing the absence of cruciate ligaments, lateral ligament insufficiency, and early knee osteoarthritis.

The tibial platform was first established; it was dysplastic and small, limiting the implant size choices for it and the femur. A starting hole was drilled, centered mediolaterally over the midpoint of the tibial canal isthmus. The tibial canal was then reamed progressively until cortical contact was achieved. The narrow tibial diaphysis and thin cortices hampered the reaming. Most importantly, the midshaft intramedullary bony bridge was very dense, leading to constitutional tibial varus and leaving no option to further reaming without the risk of tibial fracture. Using the appropriate tibial cutting guide, a proximal tibial cut 90° to the tibial mechanical axis with no tibial slope was performed. Due to multidirectional instability and the expected large flexion gap caused by the hypertrophic posterior femoral condyles, a minimal bone cut of 2 mm from the lowest point on the tibial condyle was performed. Then the tibial plate was sized, a neutral offset stem was used to connect the center of the tibial metaphysis and the varus medullary canal diaphysis until abutting the intramedullary bony bridge, and the final tibial preparation was completed.

The surgical procedure continued with an entry point made in the center of the patellar sulcus on the femoral metaphysis. The femoral reaming was done until cortical contact was achieved, but the thin femoral cortices and meta-diaphyseal incongruency made it challenging. A 6° valgus distal femoral cut was performed using the cutting block, and the femoral size was then determined. The femoral rotation was established, a non-offset femoral stem technique was decided due to the narrow canal, and the final femoral cuts were made. Balancing flexion and extension gaps were challenging. In flexion, the component was moved distally to shorten the flexion gap and allow for a reasonable liner to avoid impingement. The latter was limited by the pistol grip deformity of the femoral condyles before notching the anterior femoral cortex. The patella was not replaced. Trial implants were then used to check the knee stability and ROM. After washing and drying the bone surfaces, the femoral and tibial canals were plugged to prepare for the proper cementing technique. The final implants (femoral size E, tibial 3, polyethylene 12) were inserted after the cement was retrogradely inserted and pressurized using a third-generation cementing technique. Finally, the neurofibrotic tissue occupying the joint and around the joint space was removed to a great extent until reaching the popliteal fossa.

**Figure 3:**
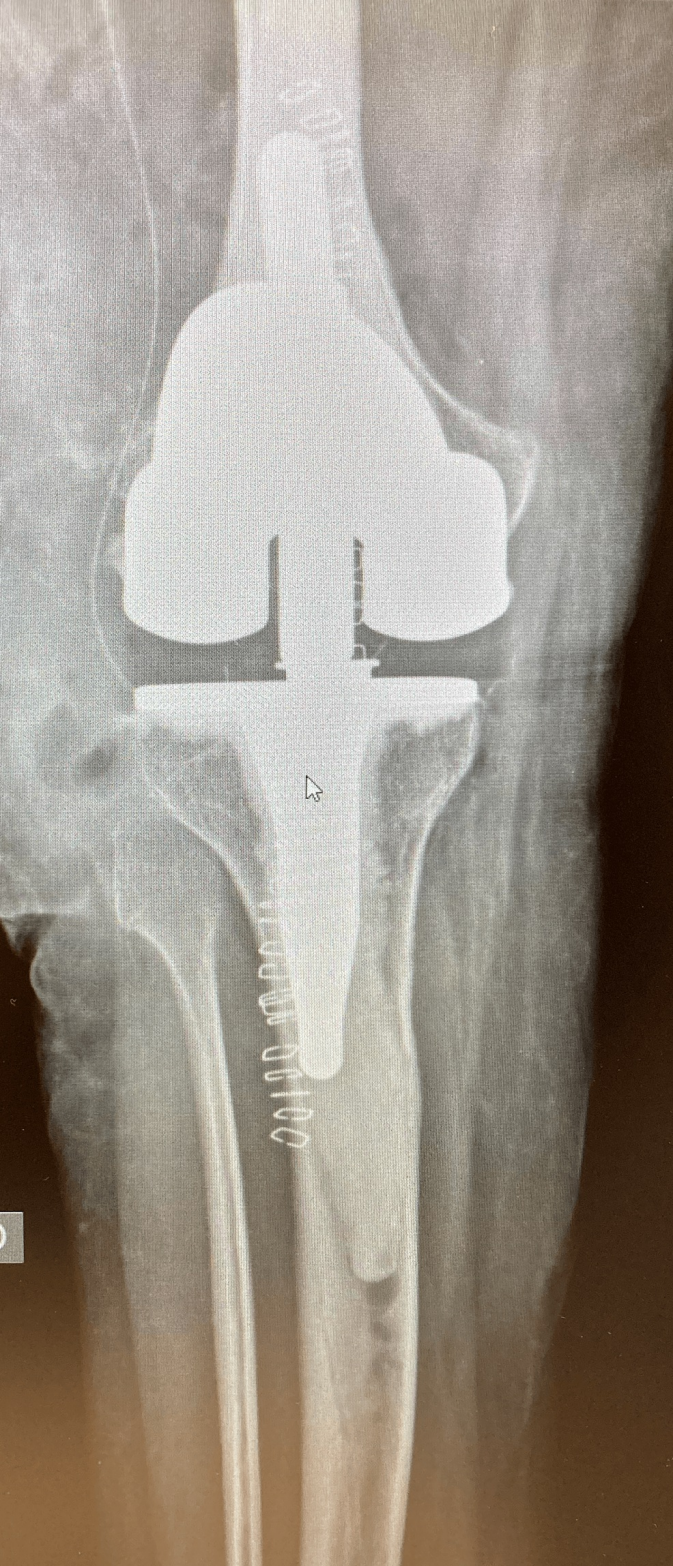
Postoperative anteroposterior knee radiograph showing the cemented rotating-hinged total knee arthroplasty.

**Figure 4:**
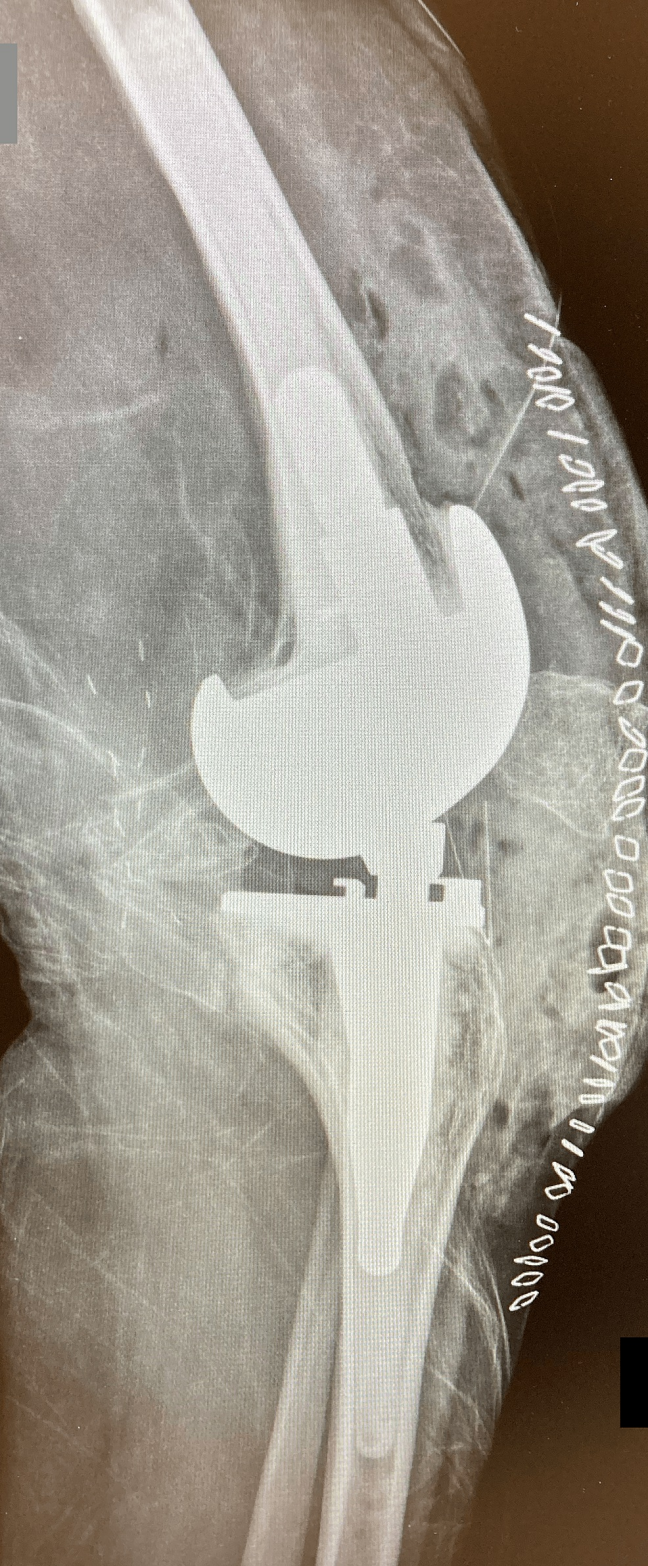
Postoperative lateral knee radiograph showing the cemented rotating-hinged total knee arthroplasty.

The patient’s recovery after surgery was uneventful, without any complications. The wound was dry, and the pain reduced gradually in the first few days. Postoperative imaging showed that the TKA prosthesis was well aligned (Figures 3, 4).

The patient could move with partial weight-bearing on the first day after surgery and was discharged from the hospital on the third day. She continued with partial weight-bearing for six weeks and then began walking as tolerated. After two months, she had no pain and could stand and walk without assistance. It has been three years since the surgery, and follow-up has been constant. Her Oxford Knee Score is 40, and she can walk with a stable knee. She can fully extend and flex her knee up to 110°, and there were no signs of implant loosening during the last follow-up visit (Video 2).

**Video 2:** The patient (on the left) is walking with a stable knee together with the doctor (on the right) three years postoperatively.

## Discussion

To our knowledge reviewing the English literature, this is the first technical report on RH-TKA being used to treat a patient with neurofibromatosis type one with a multidirectional unstable and dysplastic knee and early osteoarthritis in a very young patient. The knee instability was caused by a huge neurofibroma tumor, which pressed and deformed the underlying bony and ligamentous structures, and the neurofibromatosis disease, which affected the neuromuscular development of the joint that several reconstructive surgeries during childhood and adolescence failed to treat. This case study highlights the challenges faced in making decisions, the difficulties encountered during surgery, and the solutions implemented to overcome issues with surgical exposure, balancing, and technical problems during the complex primary TKA procedure.

The main reason for performing a TKA is to treat severe pain caused by end-stage knee arthritis [6]. However, orthopedic professionals have no agreement regarding the severity of symptoms and other indications for TKA [6]. In our case, we recommended TKA for a patient with multidirectional knee instability who had exhausted all other treatment options except for arthrodesis. It is now recognized that age is not a barrier to TKA, and the number of TKA procedures performed on younger patients is increasing [6]. The decision to undergo TKA can be challenging for the surgeon and the patient. Still, our patient was fully informed about the benefits and risks of the procedure as well as the limitations of the arthrodesis. The patient underwent conservative and limited ligamental reconstruction treatment for several years. Still, it was ineffective, proving that neurofibromatosis is recalcitrant to ligament-only reconstruction when dealing with joint instability. She also refused arthrodesis because of the potential functional limitations and the inability to switch to arthroplasty later in life [7]. Considering the surgeon’s experience and the patient’s high expectations, a decision was made to proceed with an RH-TKA.

In multidirectional instability, ligament deficiency, incongruency of the femur and tibia, femoral condyle deformity and hypoplasia, and bone defects, RH-TKA is recommended to reconstruct the unstable knee [8]. In our case, where the difference between the extension and flexion gaps was more than 10 mm, we compensated for the instability by balancing the level of bony resections and leading to a distal femoral implant position to lessen the flexion gap without using a large liner threatening patellar impingement and by tightening the extension gap to correct recurvatum.

When choosing to treat a young patient, one should consider the limitations caused by significant bony cuts and the high risk of aseptic loosening due to the level of constraint. RH-TKA is a reliable implant for complex primary TKA with inadequate collateral ligaments, significant flexion laxity, and significant bony defects [8]. Although hinged TKA implants have had high complication rates in the past, modern outcomes vary depending on the model and preoperative diagnosis [9]. Recent studies have shown that different RH-TKA models using modern fixation methods protect against aseptic loosening and improve implant longevity [10]. For this deformed and dysplastic knee with multidirectional and recurvatum instability with neurological deficit, RH-TKA was the only viable option.

During surgery, several concerns need to be addressed related to the dysplastic anatomy, including the pistol grip femoral condyles, the small lateral femoral condyle with a shallow femoral sulcus, the incongruent joint surfaces, the intramedullary tibial bone bridge leading to constitutional mid-shaft varus tibial deformity, and the unstable recurvatum alignment. Additionally, bones were small, the canals were narrow, and the bone cortices were thin due to osteoporosis but were abnormally shaped in places thick with bony bridges. These factors increased the risk of bone perforation during preparation, limiting stem extensions and offset placement. Due to the narrow canals and abnormal anatomy, we could only use short stems, and despite the patient’s young age and poor bone quality, we had to use cement. Soft tissue envelope protection was also paramount as the neurofibrous tissue was extensive, and removing it could threaten the skin and capsular tissue.

## Conclusions

To our knowledge, this is a rare technical report in the English-language literature highlighting the successful use of an RH-TKA implant in a young patient with neurofibromatosis type one and a multidirectional unstable knee with severe bony dysplasia. The procedure helped reconstruct the knee anatomy and stability, resulting in good midterm clinical outcomes without using a costly custom-made implant or a challenge regarding survival, fully hinged prosthesis.

This study showcases the obstacles in decision-making and surgery and the solutions to overcome surgical exposure, balancing, and technical problems during a complex primary TKA procedure.
